# Autophagy as a Survival Mechanism for Squamous Cell Carcinoma Cells in Endonuclease G-Mediated Apoptosis

**DOI:** 10.1371/journal.pone.0162786

**Published:** 2016-09-22

**Authors:** Atsushi Masui, Masakazu Hamada, Hiroyasu Kameyama, Ken Wakabayashi, Ayako Takasu, Tomoaki Imai, Soichi Iwai, Yoshiaki Yura

**Affiliations:** Department of Oral and Maxillofacial Surgery, Osaka University Graduate School of Dentistry, 1-8 Yamadaoka, Suita, Osaka, 565-0871, Japan; Indian Institute of Science Education and Research, INDIA

## Abstract

Safingol, L- threo-dihydrosphingosine, induces cell death in human oral squamous cell carcinoma (SCC) cells through an endonuclease G (endoG) -mediated pathway. We herein determined whether safingol induced apoptosis and autophagy in oral SCC cells. Safingol induced apoptotic cell death in oral SCC cells in a dose-dependent manner. In safingol-treated cells, microtubule-associated protein 1 light chain 3 (LC3)-I was changed to LC3-II and the cytoplasmic expression of LC3, amount of acidic vesicular organelles (AVOs) stained by acridine orange and autophagic vacuoles were increased, indicating the occurrence of autophagy. An inhibitor of autophagy, 3-methyladenine (3-MA), enhanced the suppressive effects of safingol on cell viability, and this was accompanied by an increase in the number of apoptotic cells and extent of nuclear fragmentation. The nuclear translocation of endoG was minimal at a low concentration of safingol, but markedly increased when combined with 3-MA. The suppressive effects of safingol and 3-MA on cell viability were reduced in endoG siRNA- transfected cells. The scavenging of reactive oxygen species (ROS) prevented cell death induced by the combinational treatment, whereas a pretreatment with a pan-caspase inhibitor z-VAD-fmk did not. These results indicated that safingol induced apoptosis and autophagy in SCC cells and that the suppression of autophagy by 3-MA enhanced apoptosis. Autophagy supports cell survival, but not cell death in the SCC cell system in which apoptosis occurs in an endoG-mediated manner.

## Introduction

Autophagy is originally achieved by the depletion of glucose in order to overcome starvation and promote cell survival. Although it is essentially considered to protect cellular functions [1–3], it leads to cell death under some conditions. Autophagy signals are generally mediated by the phosphatidylinositol 3-phosphase kinase (PI3k), Akt, and mammalian target of rapamycin (mTOR) signaling pathways. mTOR functions downstream of Akt and has been identified as a key regulator of autophagy [4, 5]. In normal situation, signals from growth factor receptors and nutrient sensors are collected through mTOR and its downstream effector p70 S6 kinase to promote the growth of cell and inhibit autophagy. When these signals are inhibited by pharmacological reagents or nutrient deprivation, one possible cellular response is the induction of autophagy [6]. Autophagy begins with the isolation of double-membrane-bound structures. These membrane structures elongate and microtubule-associated protein 1 light chain 3 (LC3) is recruited to the membrane [7, 8]. The elongated double membrane forms an autophagosome, which sequesters cytoplasmic proteins and organelles. Thereafter autophagosomes mature and fuse with lysosomes to become autolysosomes. The sequestered contents are then digested by lysosomal hydrolases for recycling.

Various anticancer therapies activate autophagy or autophagic cell death in cancer cells [9]. However, the autophagic response of cancer cells is not always an indication of cell death, it can be also a protective response to the treatment, allowing the recycling of proteins and cellular components. In oral squamous cell carcinoma (SCC), chemotherapeutic agents such as epigallocatechin-3, C2-ceramide, resveratrol and IL-24 that induced caspase-dependent apoptosis and/or necrosis of SCC cells also contributed to cell survival or cell death of cancer cells [10–14].

Protein kinase C (PKC) comprises a family of phospholipid-dependent serine/threonine kinases, including novel and atypical isoforms [15] and plays a pivotal role in signal transduction involved in the control of cell proliferation, differentiation and apoptosis of tumor cells including oral SCC cells [16, 17]. Safingol is a synthetic L-*thero*-dihydrosphingosine, the effects of which are attributed to its suppressive effect on the protein kinase C (PKC) family [18, 19], and its ability to affect the balance of ceramide and sphingosine 1-phosphate as a sphingosine kinase inhibitor [20]. The antitumor ability of safingol was examined in combination with doxorubicin or cisplatin in a clinical trial for solid tumors [21, 22]. We showed that safingol released the apoptogenic factor, endonuclease G (endoG), from mitochondria, which translocated into the nucleus, and this was followed by the apoptosis of human oral squamous cell carcinoma (SCC) cells at concentrations of 25 μM and 50 μM [23, 24]. Caspase 3 was not activated but the down-regulation of endoG blunted the antitumor ability of safingol under these conditions; radioactive oxygen species (ROS) were found to be upstream mediators of safingol-induced apoptosis [25, 26]. Safingol has been shown to induce autophagy by inhibiting PKCs and PI3k in human colon cancer cells, and the cell death that occurred had a distinctly autophagic morphology [6]. However, the role of autophagy in endoG-mediated apoptosis in oral SCC cells has not yet been investigated. In the present study, we examined the ability of safingol to induce autophagy and determined whether autophagy contributed to cell death or cell survival in endoG-mediated apoptosis.

## Materials and Methods

### Cells

The human oral SCC cell lines SAS and HSC-3 were obtained from the RIKEN BRC CELL BANK (Tsukuba, Japan) and Ca9-22 from the Japanese Collection of Research Bioresources (Tokyo, Japan). Cells were cultured in Dulbecco’s modified Eagle’s medium supplemented with 5% fetal bovine serum, 4 mM L-glutamine, 100 μg/ml penicillin, and 100 μg/ml streptomycin and then grown in an incubator at 37°C in a humidified atmosphere with 5% CO_2_.

### Reagents

Safingol and the broad caspase inhibitor z-VAD-fmk were obtained from Calbiochem-Novabiochem (San Diego, CA, USA) and a stock solution was made in dimetyl sulphoxide (DMSO). 3-MA, bafilomycin A1, MTT, and PI were obtained from Sigma (St.Louis, MO, USA). Acridine orange and NAC were obtained from Wako (Osaka, Japan).

### 3-(4,5-dimethylthiazol-2-yl)-2,5-diphenyltetrazolium bromide (MTT) assay

Cells were grown in 96-well culture plates and treated with safingol. Thereafter, 10 μl of 5mg/ml MTT solution was mixed with 100μl of medium in each well and cells were incubated at 37°C for 4 h. After the addition of 100 μl of 0.04N HCl in isopropanol, the plates were mixed to dissolve the dark blue crystal. The plates were read on a Benchmark Plus microplate spectrophotometer (Bio-Rad Laboratories, Hercules, CA, USA) with a reference wavelength of 630 nm and a test wavelength of 570 nm. Background absorbance at 630 nm was subtracted from the 570 nm reading as described previously [23].

### Flow cytometric analysis

FITC-annexin V and PI staining was performed using Vybrant Apoptosis Assay Kit#3 (Life Technologies, Carlsbad, CA, USA) following the manufacturer’s instructions. After being treated, floating cells were harvested with medium and attached cells were dissociated with EDTA-trypsin solution. Cells were collected by centrifugation at 1,000 rpm for 5 min. Cells were centrifuged and washed twice with phosphate-buffered saline (PBS), and the pellets were suspended in 100 μl binding buffer containing 10 mM HEPES, 140 mM NaCl, and 2.5 mM CaCl (pH 7.4) and incubated with 5 μl of FITC-annexin V and 1 μl of 100 μg/ml PI solution for 15 min at room temperature. Thereafter, 400 μl of binding buffer was added, mixed gently and kept on ice. Stained cells were analyzed by FACSCalibur (Becton Dickinson, Mountain View, CA, USA). Data were analyzed by Cell Quest software (Becton Dickinson).

Regarding acridine orange staining, cells floating and attached cells were harvested and collected by centrifugation at 1,000 rpm for 5 min. Acridine orange was added at a final concentration of 1 μg/ml and incubated for 15 min. The emission of red (564–606 nm) fluorescence was measured with a FACSCalibur.

### Transmission electron microscopy (TEM)

SAS cells were plated on 100 mm dish and cultured for 48 h. cells were treated with 10 μM safingol for 24 h and fixed in phosphate buffered 2.5% glutaraldehyde and post-fixed in 2% osmium tetra-oxide for 3 h in the ice bath. The specimens were dehydrated in a draded ethanol and embedded in the epoxy resin. Ultrathin sections obtained by ultramicrotome technique were stained with uranyl acetate for 10 min and lead staining solution for 5 min, and observed using JEM-1200 EX (JEOL, Tokyo, Japan).

### Cell fractionation

Nuclear fraction was prepared using NE-PER Nuclear and Cytoplasmic Extraction Reagents (Thermo Scientific, Waltham, MA, USA) following the manufacturer’s instructions. Cells were suspended in 500 μl of PBS in a 1.5-ml micro tube. Nuclear proteins were released from the nuclei in a high salt buffer (Nuclear Extraction Regent) and the supernatant containing nuclear proteins was obtained as the nuclear fraction after centrifugation at 15,000 x g for 10 min.

### Immunoblot analysis

To detect proteins other than cytochrome c and endonuclease G, cells were washed in PBS and lysed in a buffer containing 20 mM Tris-HCl (pH 7.4), 0.1% SDS, 1% TritonX-100, 1% sodium deoxycholate, and protease inhibitor cocktail. After sonication on ice and subsequent centrifugation at 15,000×g for 10 min at 4°C, the supernatant was collected and the protein concentration was determined using a Protein Assay Kit (Bio-Rad, Hercules, CA,USA). A protein sample (20 μg) was electrophoresed through a polyacrylamide gel and transferred to a PVDF membrane (Millipore, Bedford, MA, USA) by electroblotting. The membrane was probed with antibodies and antibody binding was detected using an enhanced chemiluminescence (ECL) kit (GE Healthcare, Amersham, Buckinghamshire, UK) according to the manufacturer’s instructions. The antibodies used were as follows: rabbit polyclonal antibodies against endoG (Sigma), mouse monoclonal antibodies against LC3 (Medical & Biological Laboratories, Nagoya, Japan), rabbit polyclonal antibodies against Atg5, beclin-1 and histone (Cell Signaling Technology, Beverly, MA, USA), mouse monoclonal antibodies against β-actin (Sigma), and horseradish peroxidase-conjugated secondary antibodies (Cell Signaling Technology, Beverly, MA, USA).

### Confocal laser microscopic analysis

After being treated, cells were fixed in 4% paraformaldehyde phosphate buffer solution (Wako) and incubated with a rabbit polyclonal antibody against endoG (Sigma, St.Louis, MO, USA) diluted 1:100 in PBS or an antibody against LC3 diluted 1:500 for 1 h at room temperature. After washing, the cells were incubated with an Alexa Fluor 488 goat anti-rabbit antibody or Alexa Fluor 488 goat anti-mouse IgG antibody (Life Technologies, Carlsbad, CA, USA) diluted 1:500 in PBS for 1 h. After washing, coverslips were mounted onto microslides using a ProLong Gold Antifate Reagent with DAPI (Life Technologies Corporation). The slides were analyzed under the confocal laser-scanning microscope Leica TCS SP8 (Leica Microsystems, Mannheim, Germany).

Regarding acridine orange staining, acridine orange was added at a final concentration of 1 μg/ml and incubated for 15 min. Cells were observed under a confocal laser microscope equipped with a 488-nm excitation filter (green fluorescence) and 579-nm excitation filter (red fluorescence).

### siRNA transfection

Chemically synthetic siRNA against endoG and AllStars negative control siRNA (nonsense siRNA) were purchased from Qiagen (Valencia, CA, USA). The target sequence of the siRNA for endoG was 5’-AAAUGCCUGGAACAACCUUGA-3’. Cells were plated on 6-well plates at a density of 1×10^5^ cells /well, cultured for 24 h, and transfected with 40 nM endoG siRNA or nonsense siRNA using Lipofectamine 2000 (Invitrogen, Carlsbad, CA, USA) according to the manufacturer’s directions. The medium was replaced with DMEM after 3 h and cells were used for experiments 24 h after transfection.

### Statistical analysis

Statistical analysis were performed using the Student’s *t*-test with Microsoft Excel (Microsoft, Redmond, WA, USA). Results were expressed as the mean±SD. Differences were considered significant at *P*<0.05.

## Results

### Inhibition of cell viability by safingol in oral SCC cells

**Fig 1:**
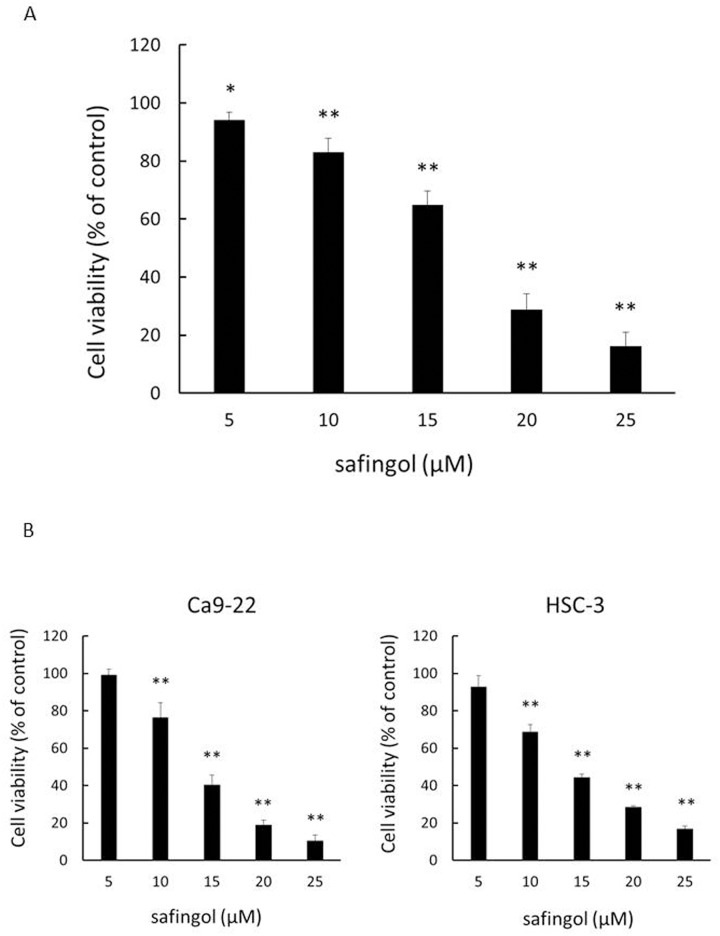
Inhibition of cell viability by safingol in oral SCC cells. (A) Oral SCC cells were treated with safingol at various concentrations for 24 h and cell viability was determined using the MTT assay. (B) The effects of safingol on the oral SCC cells lines, Ca9-22 and HSC-3, were also tested at various concentrations and cells were treated for 24 h. Data were means±SD (n = 6). *P<0.05, **P<0.01 compared to untreated control.

To determine the inhibition of cell viability by safingol, oral SCC cells were treated with various concentrations of safingol and cell viability was determined by MTT assay. When SAS cells were treated for 24 h, cell viability decreased in a dose-dependent manner (Fig 1A). Viability decreased to 83% and 16% of the control at concentrations of 10 μM and 25 μM, respectively. The decrease in cell viability by safingol was also observed in other SCC cells lines, Ca9-22 and HSC-3, in a dose-dependent manner (Fig 1B). At a concentration of 10 μM, viability decreased to 76% and 69% of the control in Ca9-22 and HSC-3 cells, respectively. The IC50 determined using the MTT assay data for SAS, Ca9-22 and HSC-3 cells were 17 μM, 15 μM and 15 μM.

### Induction of apoptosis by safingol

**Fig 2:**
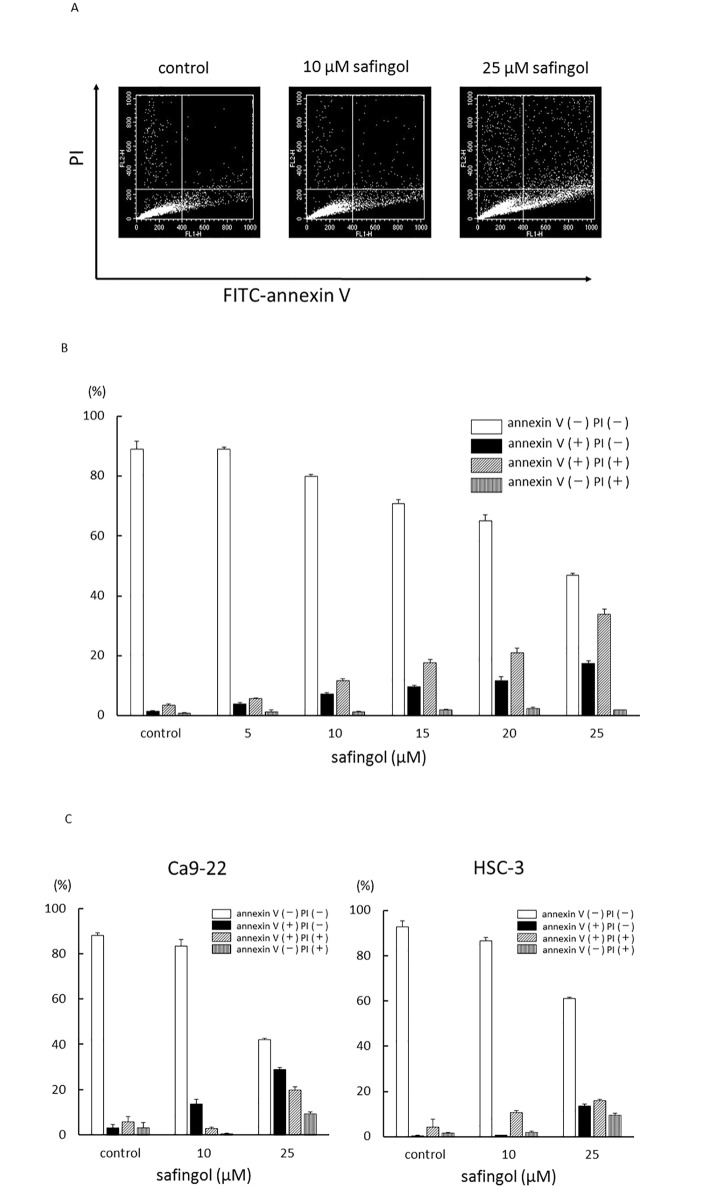
Induction of apoptosis by safingol. (A) SAS cells were treated with safingol at various concentrations for 24 h, stained with FITC-annexin V and PI, and then subjected to flow cytometry. (B) The percentages of annexin V-negative and PI-negative viable cells, annexin V- positive and PI-negative early apoptotic cells, annexin V-positive and PI-positive late apoptotic cells, and annexin V-negative and PI-positive necrotic cells were determined. (C) Ca9-22 and HSC-3 cells were also treated with safingol, stained with FITC annexin V and PI, and analyzed using flow cytometry. Data were means±SD (n = 3).

SAS cells were treated with safingol at various concentrations for 24 h, stained with annexin V conjugated to fluorescein isothiocyanate (FITC-annexin V) and propidium iodide (PI), and then subjected to flow cytometry. Cell populations were grouped into four types; annexin V-negative and PI-negative viable cells, annexin V-positive and PI-negative early apoptotic cells, annexin V-positive and PI-positive late apoptotic cells, and annexin V-negative and PI-positive necrotic cells. The number of early apoptotic cells and late apoptotic cells increased in a dose-dependent manner (Fig 2A). At all doses tested, the proportion of late apoptotic cells was also higher than that of early apoptotic cells. At concentrations of 10 μM and 25 μM, the proportions of annexin V-positive and PI-positive cells were 12% and 34%, respectively. In contrast, the number of annexin V-negative and PI-positive necrotic cells was not increased at concentrations tested (Fig 2B, S1A Table). When the effects of safingol on the cell death of Ca9-22 and HSC-3 cells were examined using flow cytometry at concentrations of 10 μM and 25 μM, increases were observed in the number of early and late apoptotic cells (Fig 2C, S1B Table).

### Induction of autophagy by safingol

**Fig 3:**
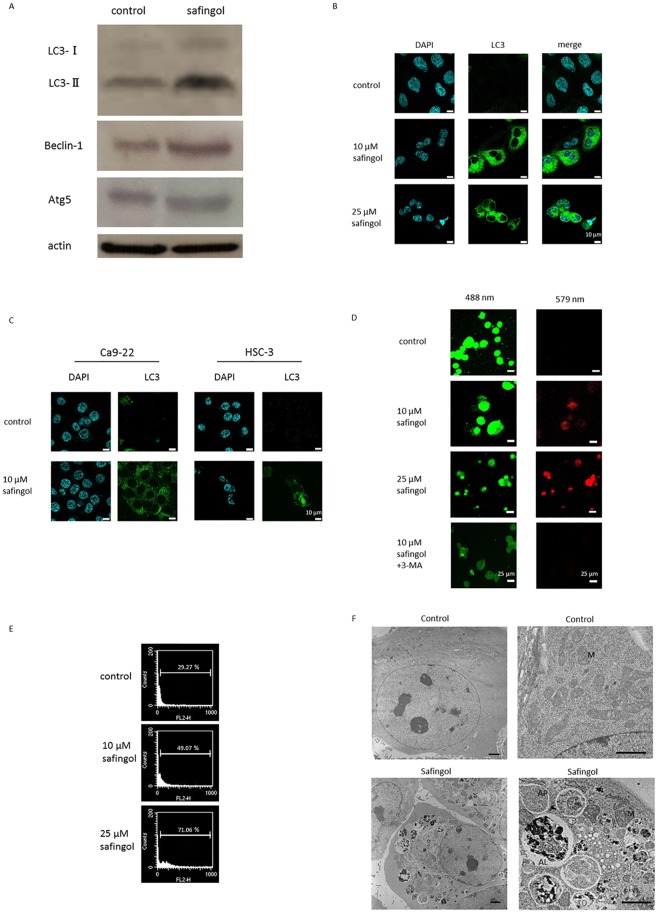
Induction of autophagy by safingol. (A) SAS cells were treated with 10 μM safingol for 24 h and the expression of LC3-I, LC3-II, Atg5 and beclin-1 were examined by immunoblotting. (B) SAS cells were treated with 10 μM or 25 μM safingol for 24 h. The expression of LC3 was examined by immunofluorescent antibody staining using an anti-LC3 antibody and DAPI. (C) Ca9-22 and HSC-3 cells were treated with 10 μM safingol for 24 h and the expression of LC3 was examined by immunofluorescent antibody staining. (D) SAS cells were treated with 10 μM or 25 μM safingol and stained with acridine orange. Green and red fluorescence was observed under a confocal laser microscope. SAS cells were also treated in combination with 10 μM safingol and 1 mM 3-MA for 24 h. (E) The emission intensity of red (564–627 nm) fluorescence in safingol-treated cells was measured by flow cytometry. (F) Representative transmission electron micrographs of SAS cells treated with 10 μM safingol for 24 h. Many vacuoles are detectable in the cytoplasm of treated cells. Higher magnification of vacuoles reveals mitochondria entrapped inside. Most cells contained intact mitochondria in untreated cells. Scale bar: 2 μm. M: mitochondria, AP: autophagosome, AL: autolysosomes.

During the activation of autophagy, LC3-I was changed to LC3-II and the proportion of LC3-II increased [8]. SAS cells were treated with safingol at a concentration of 10 μM. After being treated for 24 h, the expression of LC3-I and LC3-II was determined by an immunoblot analysis. LC3-II levels were higher after the treatment with safingol than in untreated controls. The level of beclin-1 was slightly elevated by the treatment with safingol, while there was no apparent increase in the expression of Atg5 as compared with untreated controls (Fig 3A and S1 Fig). Immunofluorescence staining using an anti-LC3 antibody and 4’,6-diamidino-2-phenylindole (DAPI) revealed the intense accumulation of LC3 in the cytoplasm of treated cells, whereas weak and diffuse cytoplasmic staining was observed in untreated cells (Fig 3B). The enhanced expression of LC3 was also demonstrated in the cytoplasm of Ca9-22 and HSC-3 cells (Fig 3C). Autophagosomes are fused to lysosomes, resulting in the production of autolysosomes, which digest the incorporated molecules. The lysosome-tropic agent, acridine orange, is a weak base that can move freely across biological membranes in an unchanged state and is characterized by green fluorescence. The protonated form of acridine orange was previously shown to accumulate in acidic compartments and formed aggregates that were characterized by red fluorescence [27, 28]. After the treatment of cells with 10 μM or 25 μM safingol for 24 h, red fluorescence was more intense in treated cells than in untreated controls, which were mostly stained green (Fig 3D). The emission of red (564–627 nm) fluorescence was quantified using flow cytometry and was found to be 20% higher in safingol-treated cells than in untreated cells (Fig 3E).

The presence of autophagic vesicles was further confirmed at the ultrastructural level through a transmission electron microscope. Most SAS cells contained intact mitochondria distributing throughout the homogeneous cytoplasm in untreated cells. When cells were treated with 10 μM safingol for 24 h, intracellular space was occupied by large vascular structures. They contained degraded mitochondria and high-density structures, which were considered to be autophagosomes and/or autolysosomes. Degraded mitochondria were also observed in the cytoplasm (Fig 3F).

### Augmentation of cell death by autophagy inhibitors

**Fig 4:**
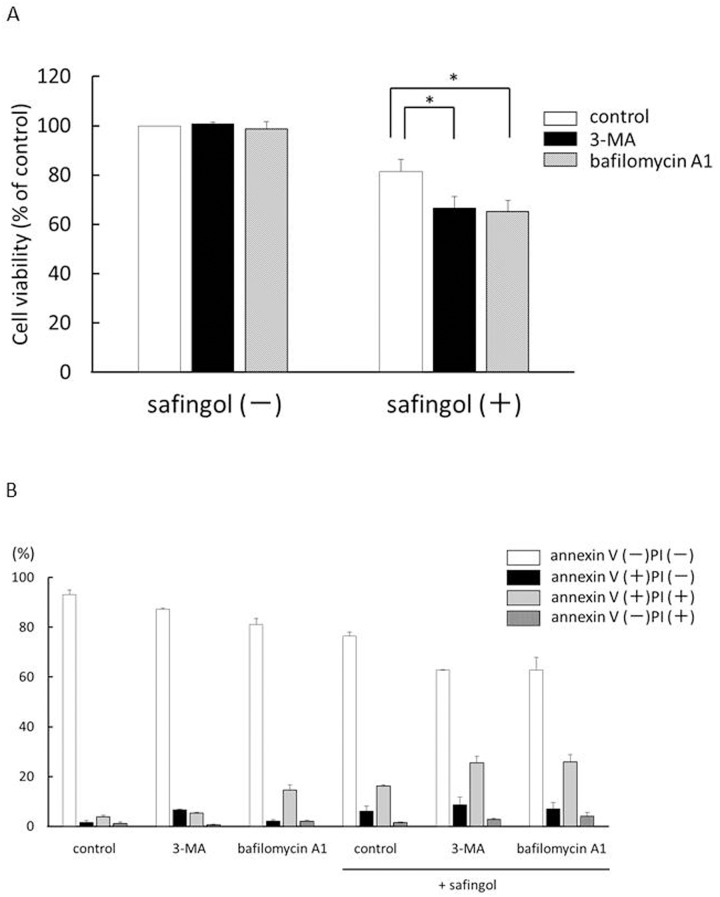
Augmentation of cell death by autophagy inhibitors. (A) SAS cells were treated with 10 μM safingol and 1 mM 3-MA or 5 nM bafilomycin A1 for 24 h and cell viability was measured by the MTT assay. Data were means±SD (n = 3), *P<0.05. (B) SAS treated with safingol and 3-MA or bafilomycin A1 for 24 h were analyzed using flow cytometry and the proportion of viable cells, early apoptotic cells, late apoptotic cells, and necrotic cells were determined.

3-metyladenine (3-MA) is a known inhibitor of autophagosome formation while bafilomycin A1 inhibits the formation of autophagosomes and autolysosomes [29, 30]. 3-MA and bafilomycin A1 were used at concentrations that did not suppress cell viability. Although 10 μM safingol decreased cell viability to 84% of the control, the addition of 1 mM 3-MA or 5 nM bafilomycin A1 further decreased cell viability; cell viability became 72% and 70% of the control, respectively. A significant difference (*P*<0.01) was observed between safingol alone and the combination with 3-MA or bafilomycin A1 (Fig 4A). The effect of autophagy inhibitors was observed in Ca9-22 and HSC-3 cells, though the difference was not significant in Ca9-22 cells (S2 Fig). Since the effect of autophagy inhibitors was clear in SAS cells, the following analyses were performed using this cell lines.

To determine whether decreases in cell viability were due to apoptosis, SAS cells were treated with a combination of safingol and 3-MA or bafilomycin A1 and were subjected to flow cytometry. In combination with 3-MA, the proportion of annexin V-positive and PI-positive cells increased from 16 to 24%, indicating an increase in the number of apoptotic cells (Fig 4B). Bafilomycin A1 also increased the proportion of apoptotic cells. Nuclear staining with Hoechst 33342 revealed the occurrence of nuclear fragmentation in SAS cells treated with safingol and 3-MA for 24 h (S3 Fig). The inhibitory effects of 3-MA on the formation of autolysosomes were examined using acridine orange staining. In the presence of 3-MA, red fluorescence in safingol-treated cells became weak and the intensity of red fluorescence was reduced from 44% to 17%, indicating a decrease in autophagy by 3-MA (Fig 3D).

### Nuclear translocation of endoG by safingol and 3-MA

**Fig 5:**
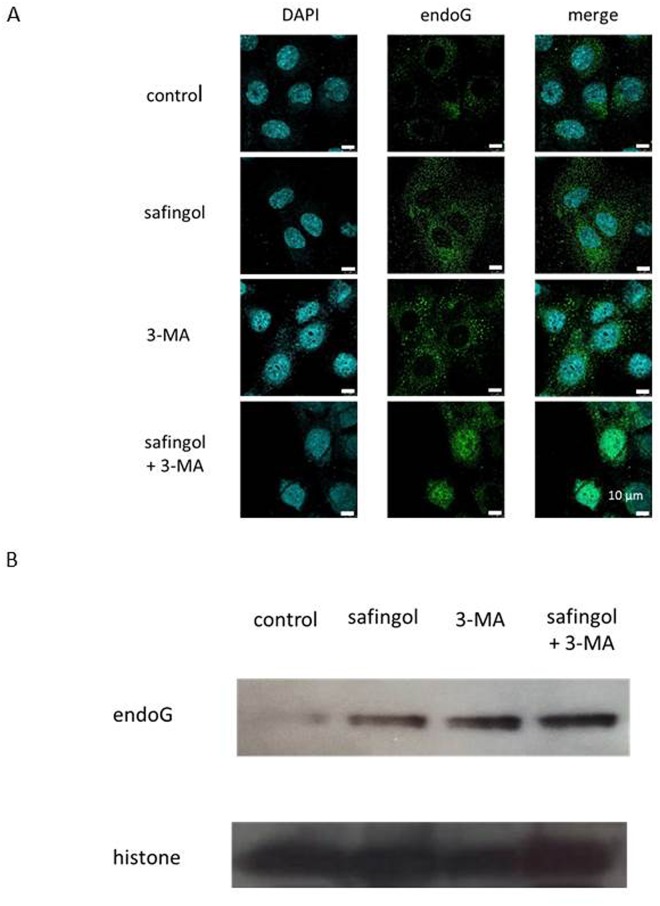
Translocation of endoG by safingol and 3-MA. (A) SAS cells were treated with 10 μM safingol and 1 mM 3-MA for 24 h and the localization of endoG was examined by immunofluorescent antibody staining. (B) SAS cells were treated with safingol with or without 3-MA for 24 h and proteins in nuclear fraction was examined for the expression of endoG using an immunoblot analysis.

Safingol was previously shown to induce the translocation of endoG from mitochondria as well as the apoptosis of oral SCC cells at concentrations of 25 μM and 50 μM [23, 24]. When SAS cells were treated with 10 μM safingol for 24 h and examined by immunofluorescent antibody staining, endoG was observed in the cytoplasm diffusely and nuclear accumulation was not observed. The treatment with 3-MA alone did not induce the nuclear translocation of endoG; however, nuclear localization was detected after the treatment with safingol and 3-MA for 24 h (Fig 5A).

Nuclear fraction was prepared to clarify the translocation of endoG from mitochondria to the nucleus and the expression of endoG was examined by an immunoblot analysis. The treatment with safingol was insufficient to induce the translocation of endoG at this concentration; however, in the presence of 3-MA, endoG was increased in the nuclear fraction (Fig 5B and S4 Fig). Histone was used as a nuclear marker.

### Involvement of endoG in safingol and 3-MA-induced cell death

**Fig 6:**
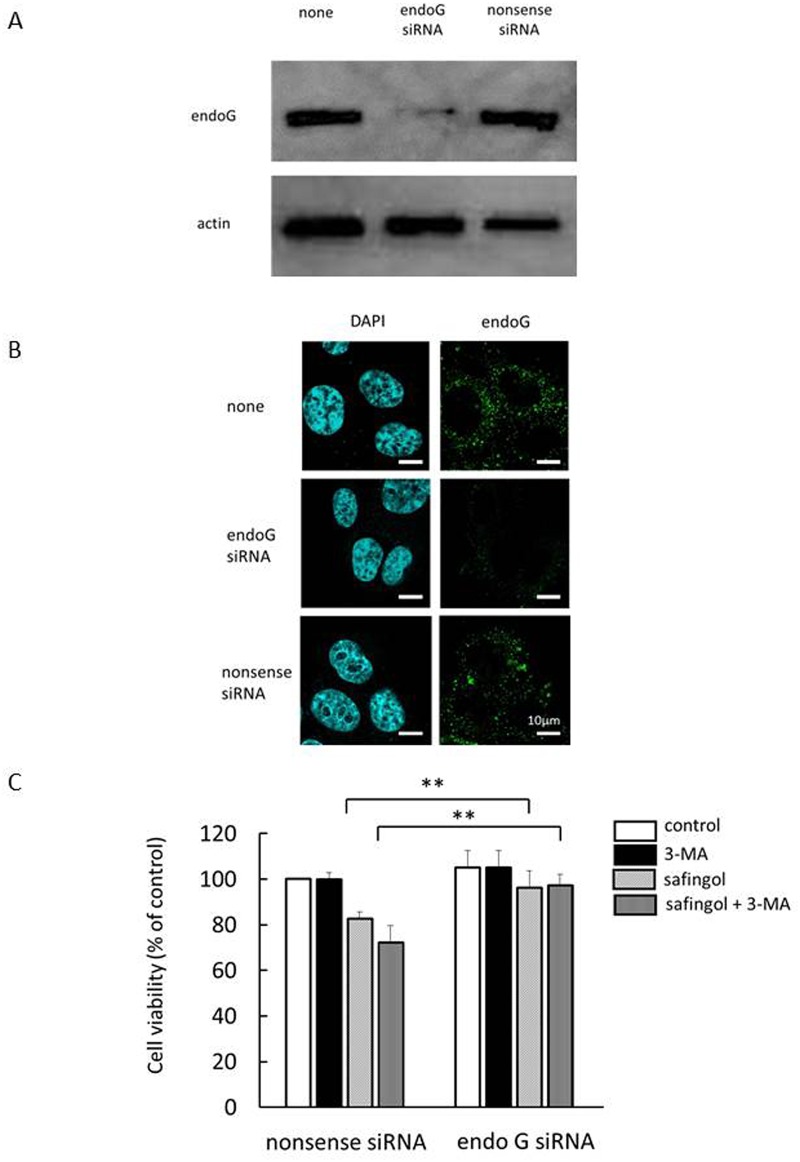
Involvement of endoG in safingol and 3-MA-induced cell death. (A) SAS cells were transfected by either endoG-siRNA or nonsense siRNA. Twenty-four hours after transfection, the expression of endoG was examined in the cells by an immunoblot analysis. (B) The expression of endoG in si-RNA-transfected cells was examined by immunofluorescent antibody staining. (C) si-RNA-transfected cells were treated with 10 μM safingol with or without 3-MA for 24 h and cell viability was measured by the MTT assay. Data were means±SD (n = 6), **P<0.01.

The effects of the knockdown of endoG on cell death were examined. SAS cells were transfected with endoG siRNA or nonsense siRNA and examined by an immunoblot analysis and immunofluorescent antibody staining. The expression of endoG was markedly decreased in endoG siRNA-transfected cells (Fig 6A and 6B and S5 Fig). When nonsense siRNA-transfected cells were treated with safingol or a combination of safingol and 3-MA, a suppressive effect was observed, but the combined effect on cell viability was diminished in endoG siRNA-transfected cells (Fig 6C); a significant difference (*P*<0.01) was observed between nonsense-siRNA-transfected cells and siRNA-transfected cells.

### Absence of pan-caspase inhibitory effects

**Fig 7:**
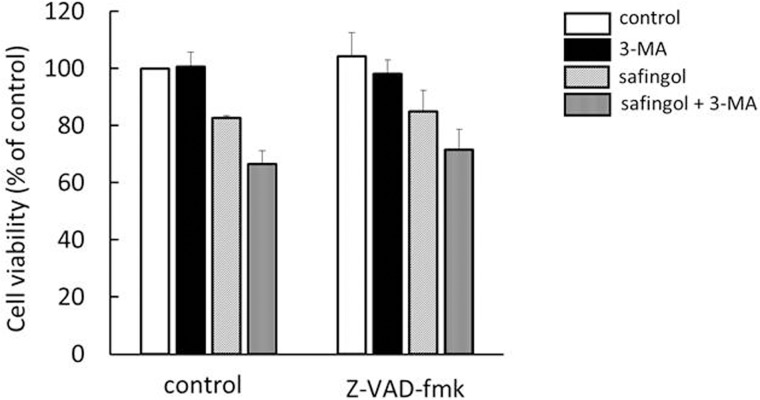
Absence of the effect of a caspase inhibitor on the cytotoxic effect of safingol and 3-MA. SAS cells were pretreated with the pan-caspase inhibitor, z-VAD-fmk for 2 h, and then treated with safingol and 3-MA in the absence of z-VAD-fmk for 24 h. Cell viability was determined by the MTT assay. There was no significant difference between control and z-VAD-fmk-treated group. Data were means±SD (n = 3).

The role of caspase-dependent cell death was examined. SAS cells were pretreated with the pan-caspase inhibitor z-VAD-fmk and then treated with 10 μM safingol and 1 mM 3-MA in the absence of z-VAD-fmk for 24 h. The caspase inhibitor did not have a suppressive effect on the cytotoxicity of the combined treatment with 3-MA. There was no significant difference between each control and z-VAD-fmk-treated group (Fig 7).

### Suppression of cell death by the ROS scavenger NAC

**Fig 8:**
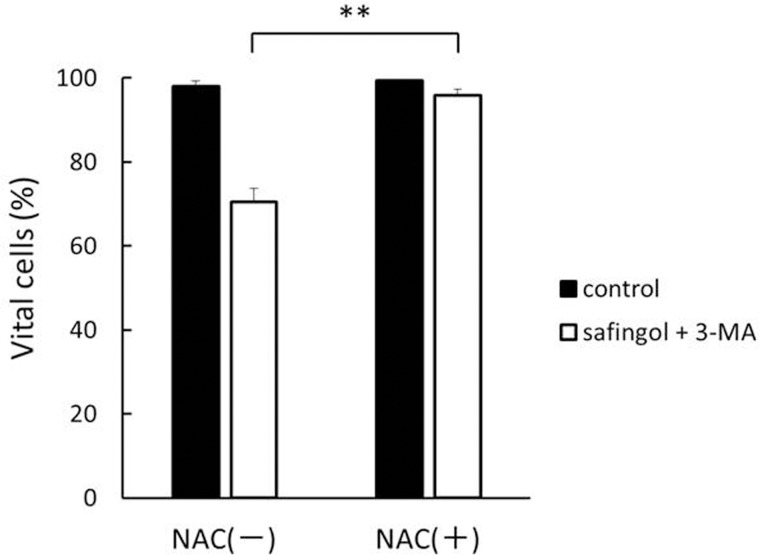
Suppression of cell death by the ROS scavenger, NAC. SAS cells were pretreated with NAC for 2 h and then treated with safingol and 3-MA in the presence of NAC for 24 h. Cell viability was determined by the MTT assay. Data were means±SD (n = 3), **P<0.01.

We previously reported that the treatment of SAS cells with 15 μM safingol produced ROS and also that the ROS scavenger, N-acetyl-L-cysteine (NAC), could prevent apoptosis, suggesting ROS as an upstream factor in the endoG-mediated pathway [25]. SAS cells were treated with safingol and 3-MA in the presence of NAC for 24 h and analyzed using flow cytometry. The increase induced in the number of late apoptotic cells by the combined treatment was markedly reduced (Fig 8), indicating the important role of ROS in cell death.

## Discussion

Apoptosis and necrosis are well-known mechanisms underlying cell death induced by anticancer therapies. However, emerging studies have demonstrated the existence of a non-apoptotic form of programmed cell death called autophagic cell death [31]. Autophagic cell death and apoptosis are different in their morphology and biochemical characters. Caspases are not activated in autophagic cell death, unlike apoptotic cell death and neither DNA degradation nor nuclear fragmentation is apparent. Caspase-independent cell death, with an increase in the number of autophagic vacuoles may be specific in autophagic cell death [32].

Response to many anticancer drugs, autophagic cell death occurred in cancer cells derived from the breast, colon, prostate, and brain [9, 32]. Tamoxifen, targets the estrogen receptor and induces cell death of breast cancer with autophagy by down-regulating the expression of protein kinase B/Akt [32–34]. Rapamycin, an inhibitor of mTOR, induces autophagy and also suppresses the proliferation of malignant glioma cells [35]. The inhibition of anti-autophagic proteins, such as Bcl2, PKCδ, and tissue transglutaminase 2 (TG2), may lead to autophagic cell death in some apoptosis-resistant cancers [36].

A number of sphingolipids including safingol have been implicated in the induction of autophagy [37]. Coward et al. [6] treated colon cancer cells with 12 μM safingol and found that safingol inhibited PKCβ1, PKCδ, and PKCε, and also inhibited the phosphorylation of the PI3k/Akt/mTOR pathway and mitogen-activated protein kinase (MAPK) pathway. Cell death caused by safingol had a distinctly autophagic morphology and biochemical signature. Ling et al. [26] reported that a long-term treatment with 5 μM safingol induced accidental necrotic cell death, but not apoptosis in human colon cancer and breast cancer cells.

We previously reported that safingol at concentrations of 25 μM and 50 μM induced apoptosis in oral SCC cells in which endoG was released from mitochondria and translocated to the nucleus to fragment chromosome DNA [23, 24]. In the present study, we tested the cytotoxic ability of safingol at different concentrations in oral SCC cell lines including SAS, Ca9-22, and HSC-3 cells. Consistent with previous findings, we demonstrated that the effects of safingol on cell viability were concentration-dependent. Cell viability was markedly decreased at a safingol concentration of 25 μM in all of the cell lines tested, whereas cell death was marginal at 10 μM safingol in oral SCC cells. Using flow cytometry, we found that the numbers of annexin V- and PI-positive cells as well as annexinV-positive and PI-negative cells were increased at 10 μM and 25 μM, whereas the number of annexin V-negative and PI- positive necrotic cells was not, indicating that safingol could induce apoptotic cell death.

We found that LC3-II was expressed after the treatment of SAS cells with safingol and LC3 was observed in the cytoplasm by immunofluorescent antibody staining. Furthermore, the safingol treatment increased AVOs that were produced by fusing autophagosomes with lysosomes and emitted red fluorescence after acridine orange staining. Furthermore, transmission electron microscopy revealed autophagic vacuoles containing degraded mitochondria in the cytoplasm of treated cells, indicating that safingol induced autophagy at the concentration of 10 μM.

To study the role of autophagy in response to therapy, autophagy inhibitors have been developed. The PI3K class-III inhibitor 3-MA can inhibit pre-autophagosome formation and prevent the cell death by tamoxifen, indicating that autophagy may be responsible for the antitumor action of this drug [32, 33]. Bafilomycin A1 and hydroxychloroquine inhibit autophagy by preventing the fusion of autophagosomes and lysosomes. [2] Miki et al. [38] reported that the inhibition of autophagy by 3-MA lowered resveratrol-induced cytotoxicity by decreasing caspase-8 and caspase-3 levels, indicating the function of autophagy as a cell death mechanism. Similarly, both pharmacological and genetic inhibition of autophagy enhanced the resveratrol-induced cytotoxicity to the human esophageal SCC cells [39]. On the other hand, the inhibition of autophagy in cancer cells may be therapeutically beneficial under certain conditions because it can sensitize cancer cells to different therapeutics, including DNA-damaging agents, anti-hormone therapies, and radiation therapy [28, 40, 41]. Zhu et al. [42] showed that salinomycin induced both apoptosis and autophagy in osteoblastoma cells. The inhibition of autophagy by 3-MA or the RNA interference of LC3B enhanced salinomycin-induced cytotoxicity and caspase-dependent apoptosis. Ling et al. [26] indicated that autophagy protected cells from safingol-induced and reactive oxygen species (ROS)-mediated necrosis using human breast cancer and colorectal cancer cells, as the suppression of autophagy by 3-MA or bafilomycin A1 significantly augmented cell death caused by safingol, but the involvement of endoG-mediated apoptosis was not investigated.

The role of autophagy in endoG-mediated cell death had not yet been investigated. We found that the cytotoxic effects of safingol were further enhanced by 3-MA, even if the inhibitor itself did not affect cell viability. The decrease observed in cell viability was attributed to an increase in the number of apoptotic cells based on the finding of a flow cytometric analysis and nuclear staining with Hoechst 33342. We also demonstrated that endoG was essential for cell death when safingol and 3-MA were combined, because endoG translocated from mitochondria to the nucleus during cell death, and cytotoxic effects were abolished by the down-regulation of endoG by siRNA transfection. Even if the caspase signal pathway was blocked using a pan-caspase inhibitor, the cytotoxic effects of safingol and 3-MA were not affected. Thus, cell death induced by safingol and the inhibition of autophagy was considered to be mediated through a caspase-independent and endoG-dependent pathway. ROS may mediate the induction of apoptosis and/or autophagy in several types of cancer cells [26, 38, 43]. A previous study showed that ROS production was involved in the safingol induced-apoptosis of oral SCC cells [25]. In the present study, the ROS scavenger NAC prevented the induction of cell death caused by the combination of safingol and 3-MA. This result indicated that ROS production was essential as an upstream effector in the endoG-mediated apoptosis of SCC cells.

We concluded that safingol induced apoptosis and autophagy in human oral SCC cells, in which autophagy played a protective role in endoG-mediated apoptosis, but did not induce autophagic cell death. To our knowledge, this is the first report that indicated the protective role of autophagy in safingol-induced and endoG-mediated apoptosis of oral SCC cells. Several *in vitro* and *in vivo* studies demonstrated that safingol augmented the efficacy of other chemotherapeutic agents, including fenretinide, irinotecan, mitomycin-C, and cisplatin in various tumors [16, 20, 44]. The inhibitory effects of other anticancer agents on autophagy must be considered when they are used in combination with safingol in clinical trials.

## Supporting Information

S1 Table (A) The raw data presented in Fig 2B. (B) The raw data presented in Fig 2C.
(TIF)

S1 Fig Complete scan of the blots presented in Fig 3A. (TIF)

S2 Fig The effect of autophagy inhibitors was observed in Ca9-22 and HSC-3 cells. (TIF)

S3 Fig SAS cells were treated with safingol and 3-MA for 24 h and dissociated with EDTA-trypsin, and nuclei were stained using Hoechst 33342. Arrows indicate apoptotic cells.
(TIF)

S4 Fig Complete scan of the blots presented in Fig 5B. (TIF)

S5 Fig Complete scan of the blots presented in Fig 6A. (TIF)
