# Supplementary figures and images for: Autophagy as a Survival Mechanism for Squamous Cell Carcinoma Cells in Endonuclease G-Mediated Apoptosis
Source: PLoS One. 2016 Sep 22;11(9):e0162786. doi: 10.1371/journal.pone.0162786 (PMC5033396; doi:10.1371/journal.pone.0162786)

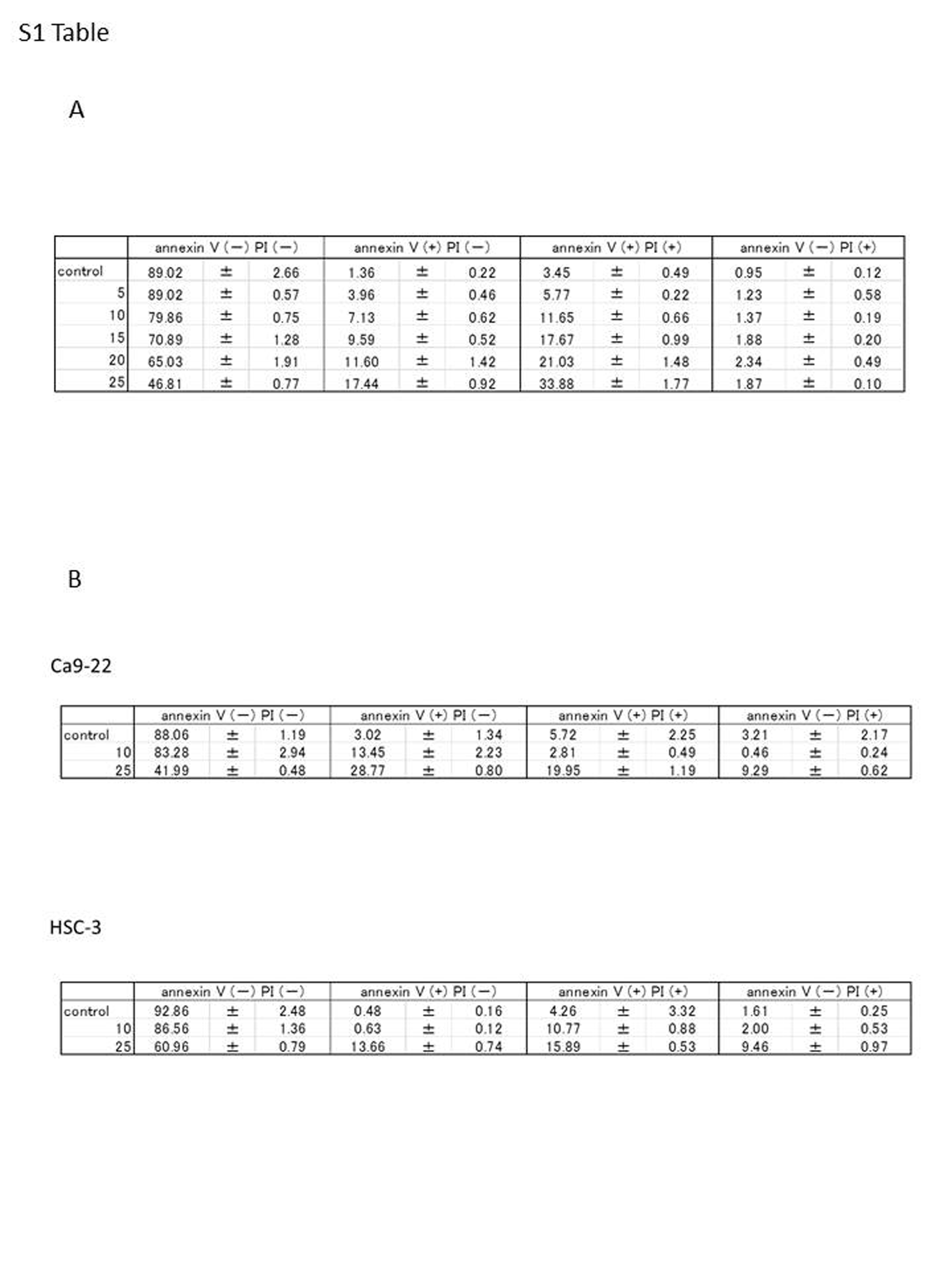

Supplement: S1 Table — (A) The raw data presented in Fig 2B. (B) The raw data presented in Fig 2C. (TIF) [file pone.0162786.s001.tif]

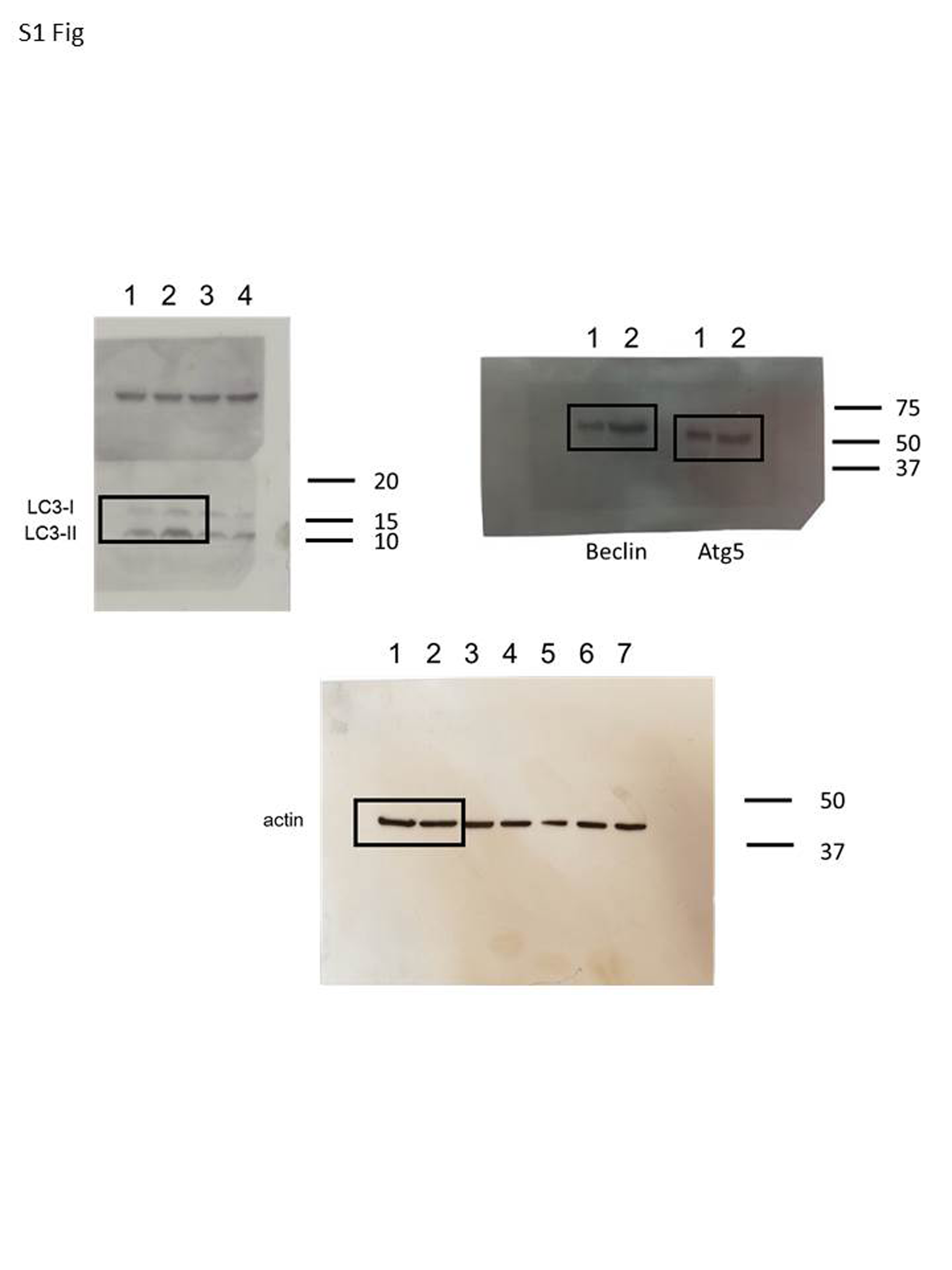

Supplement: S1 Fig — (TIF) [file pone.0162786.s002.tif]

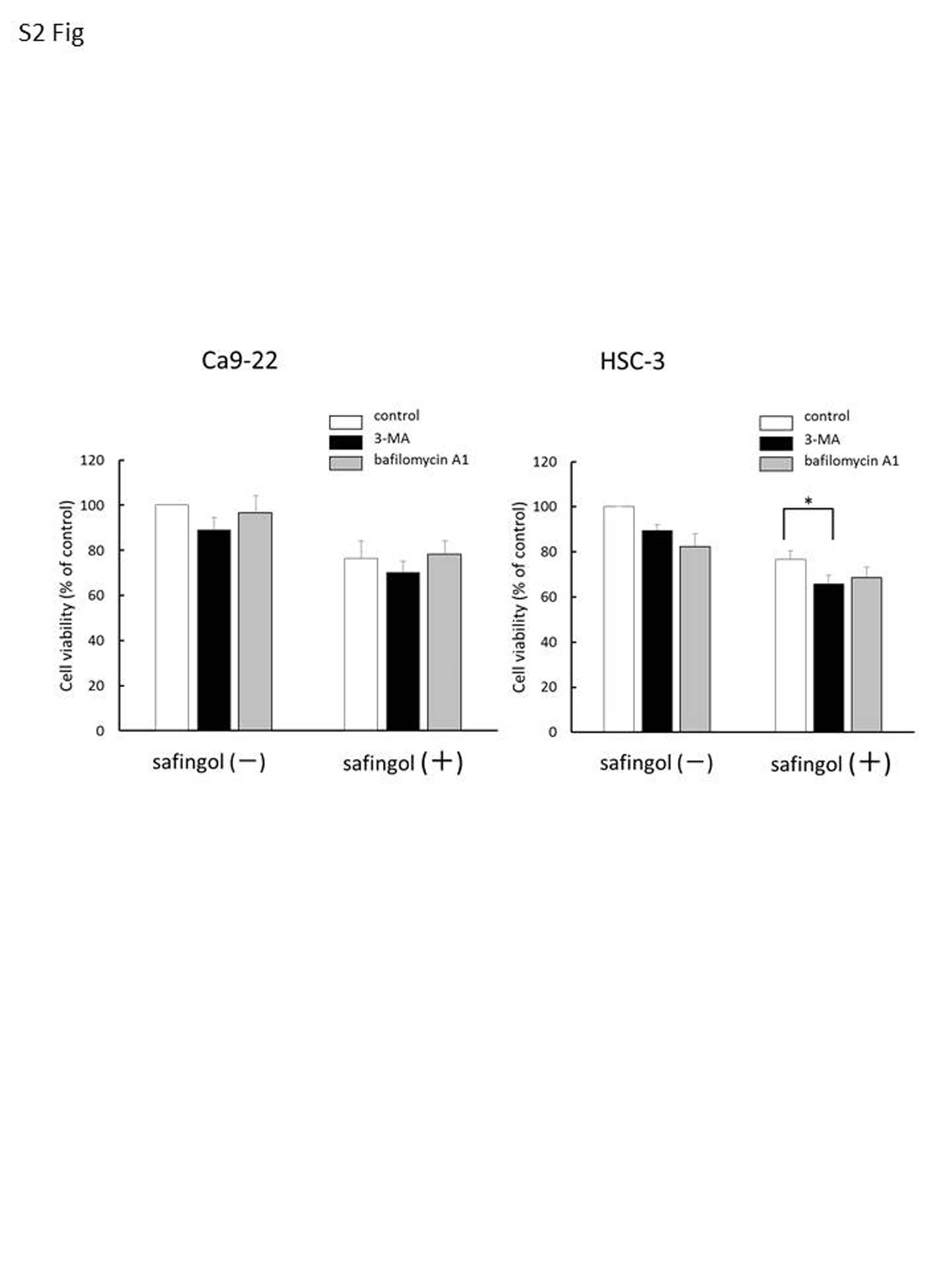

Supplement: S2 Fig — (TIF) [file pone.0162786.s003.tif]

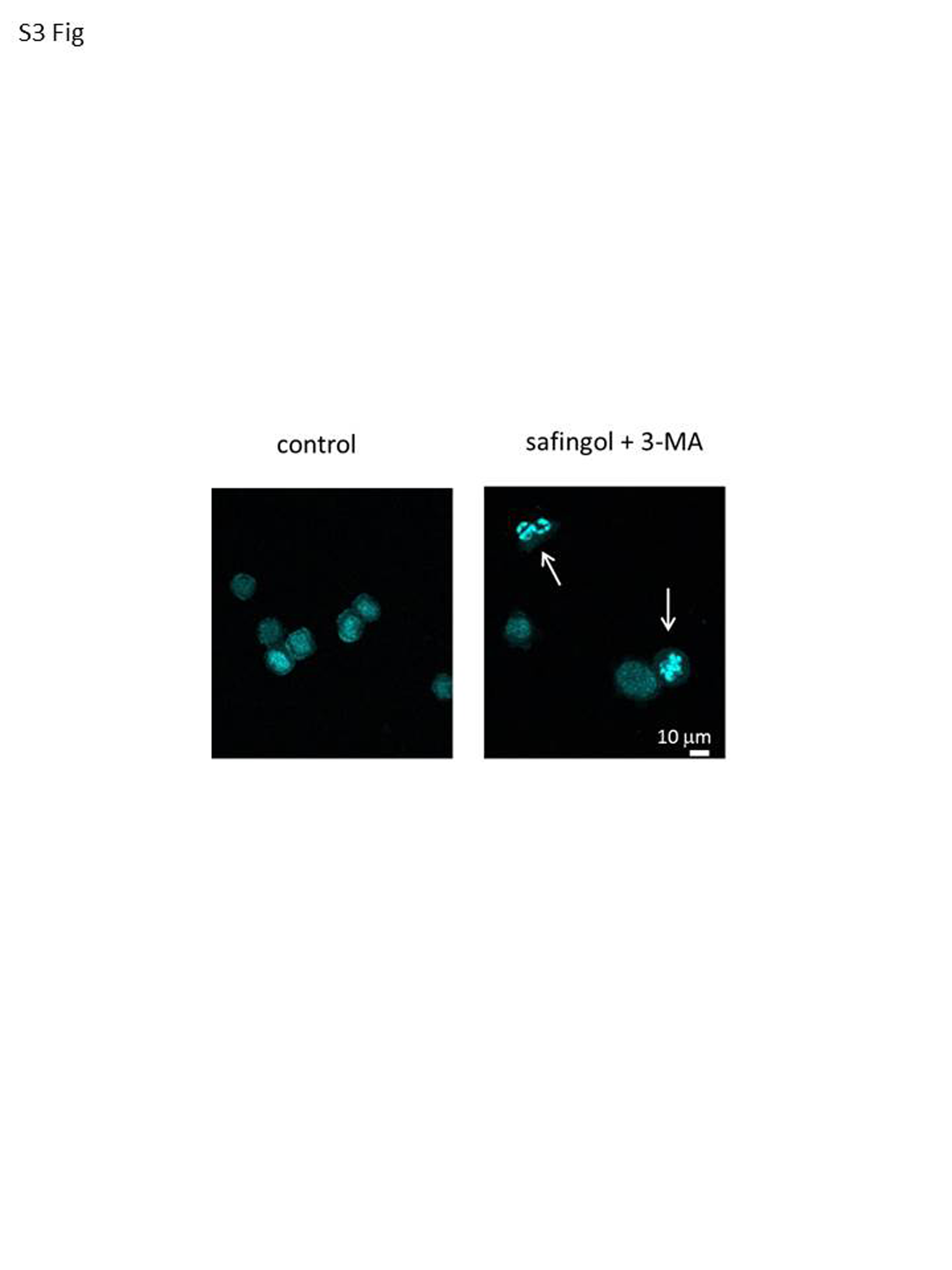

Supplement: S3 Fig — Arrows indicate apoptotic cells. (TIF) [file pone.0162786.s004.tif]

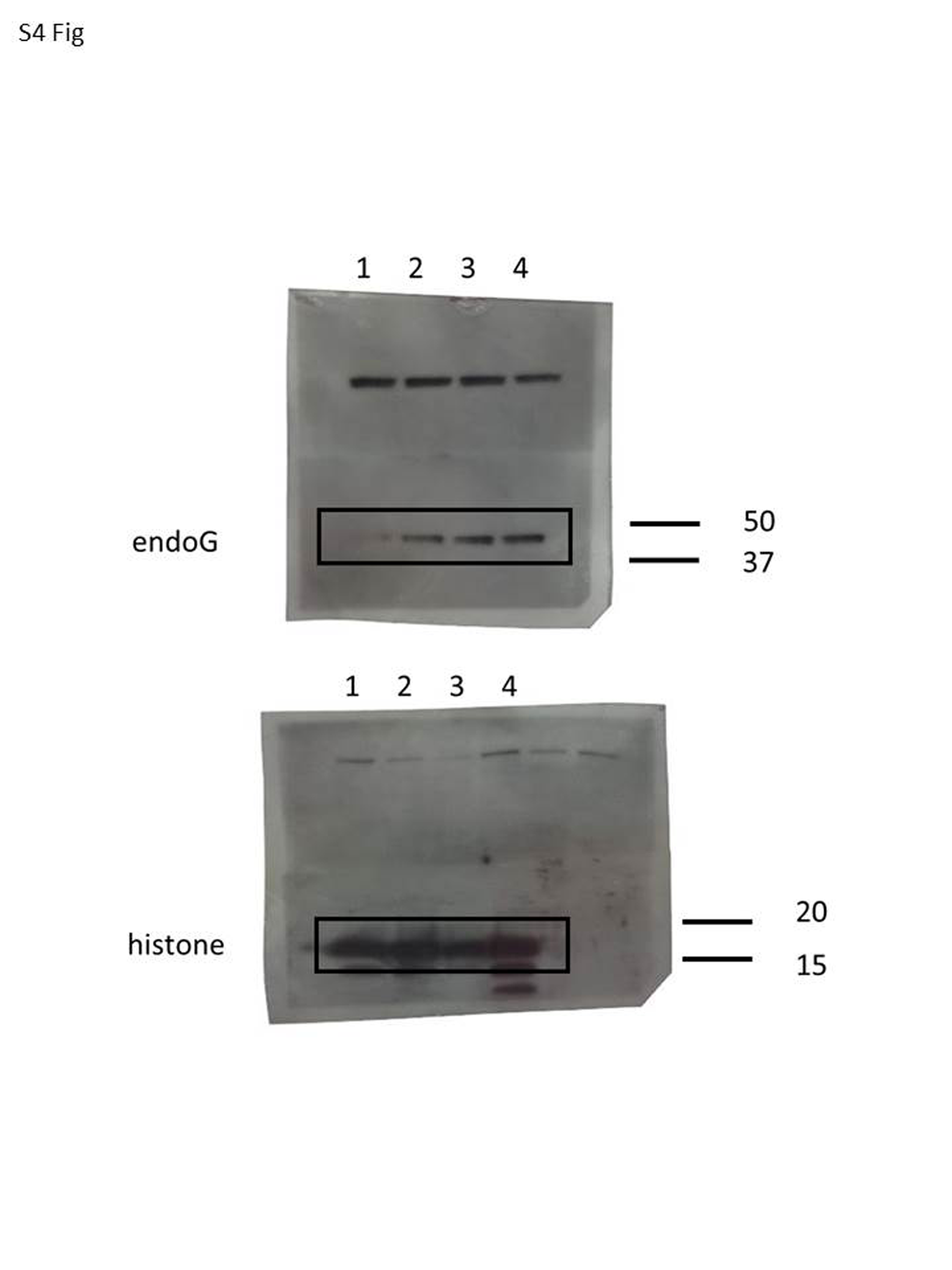

Supplement: S4 Fig — (TIF) [file pone.0162786.s005.tif]

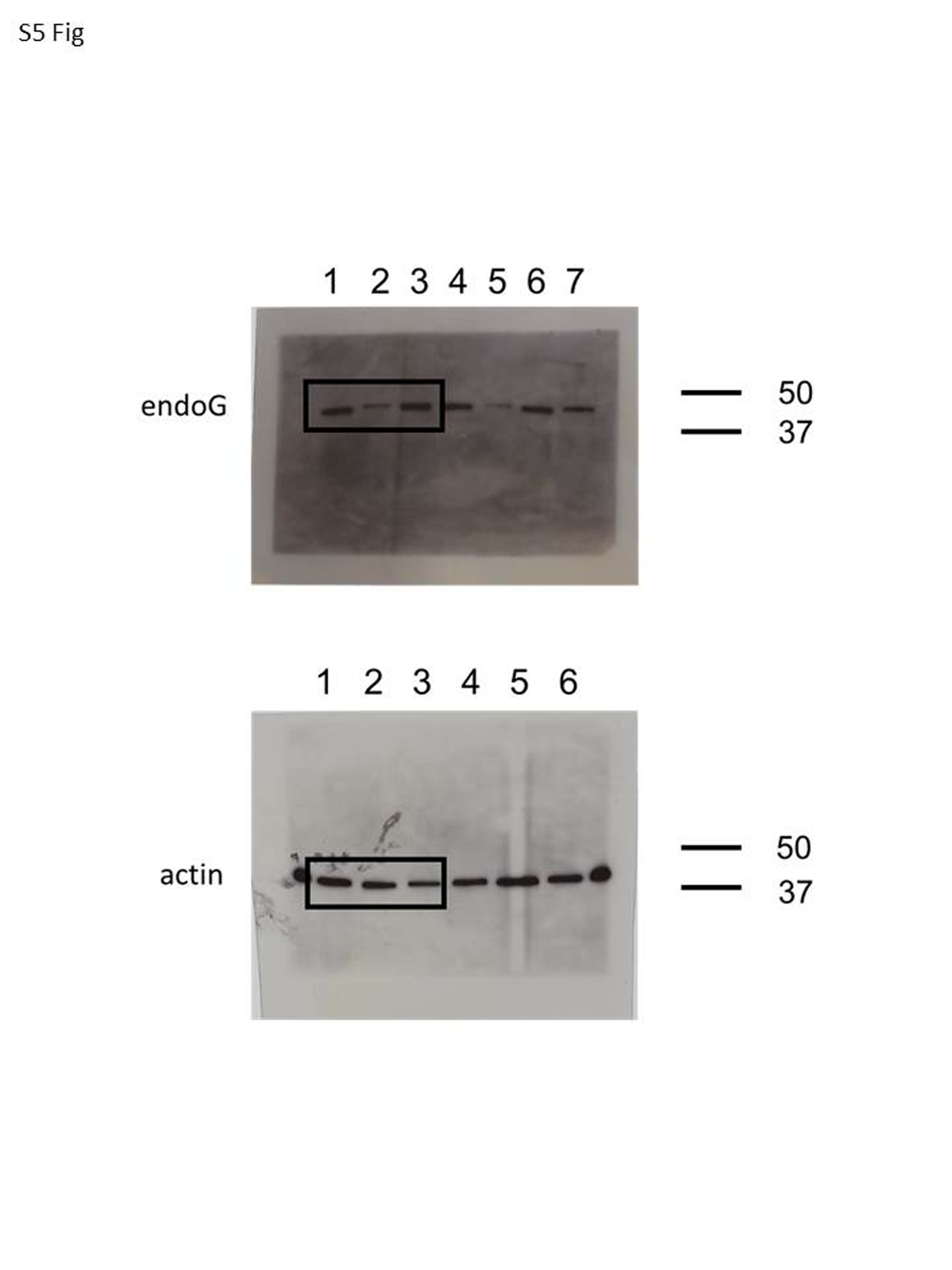

Supplement: S5 Fig — (TIF) [file pone.0162786.s006.tif]
